# Identification of virion-associated transcriptional transactivator (VATT) of SGIV ICP46 promoter and their binding site on promoter

**DOI:** 10.1186/s12985-019-1210-0

**Published:** 2019-09-03

**Authors:** Li-Qun Xia, Jian-Lin Chen, Hong-Lian Zhang, Jia Cai, Sheng Zhou, Yi-Shan Lu

**Affiliations:** 1 Shenzhen Institute of Guangdong Ocean University, Shenzhen City, Guangdong China; 2 College of Fisheries, Guangdong Ocean University, Zhanjiang City, Guangdong China; 3 Guangdong Provincial Engineering Research Center for Aquatic Animal Health Assessment, Shenzhen Public Service Platform for Evaluation of Marine Economic Animal Seedings, Shenzhen City, Guangdong China; 4 Guangdong Provincial Key Laboratory of Pathogenic Biology and Epidemiology for Aquatic Economic Animal, Guangdong Ocean University, Zhanjiang City, Guangdong China; 5 College of Marine Sciences, South China Agricultural University, Guangzhou City, Guangdong China

**Keywords:** Singapore grouper iridovirus (SGIV), ICP46, Promoter, Virion-associated transcriptional transactivator (VATT), Downstream promoter element (DPE)

## Abstract

**Background:**

Iridoviruses are large DNA viruses that cause diseases in fish, amphibians and insects. Singapore grouper iridovirus (SGIV) is isolated from cultured grouper and characterized as a ranavirus. ICP46 is defined to be a core gene of the family Iridoviridae and SGIV ICP46 was demonstrated to be an immediate-early (IE) gene associated with cell growth control and could contribute to virus replication in previous research.

**Methods:**

The transcription start site (TSS) and 5′-untranslated region (5′-UTR) of SGIV ICP46 were determined using 5′ RACE. The core promoter elements of ICP46s were analyzed by bioinformatics analysis. The core promoter region and the regulation model of SGIV ICP46 promoter were revealed by the construction of serially deleted promoter plasmids, transfections, drug treat and luciferase reporter assays. The identification of virion-associated transcriptional transactivator (VATT) that interact with SGIV ICP46 promoter and their binding site on promoter were performed by electrophoretic mobility shift assays (EMSA), DNA pull-down assays and mass spectrometry (MS).

**Results:**

SGIV ICP46 was found to have short 5′-UTR and a presumptive downstream promoter element (DPE), AGACA, which locates at + 36 to + 39 nt downstream of the TSS. The core promoter region of SGIV ICP46 located from − 22 to + 42 nt relative to the TSS. VATTs were involved in the promoter activation of SGIV ICP46 and further identified to be VP12, VP39, VP57 and MCP. A 10-base DNA sequence “ATGGCTTTCG” between the TSS and presumptive DPE was determined to be the binding site of the VATTs.

**Conclusion:**

Our study showed that four VAATs (VP12, VP39, VP57 and MCP) might bind with the SGIV ICP46 promoter and be involved in the promoter activation. Further, the binding site of the VATTs on promoter was a 10-base DNA sequence between the TSS and presumptive DPE.

## Background

Iridoviruses are large DNA viruses that cause diseases in fish, amphibians, reptiles and insects, and result in significant economic and ecological losses [1, 2]. Similar to other large dsDNA viruses, the gene transcription of iridoviruses can be divided into 3 categories according to the sequence: immediate-early (IE), early (E) and late (L), their corresponding protein encoding are very early protein, early protein and late protein [3–5]. As the first step of viral temporal expression, IE genes are the earliest genes expressed in the cells after virus infection, and the transcription of IE genes are essential for the ordered cascade of viral life events, such as manipulating the transcription of E genes and L genes, affecting the expression of host genes, controlling cell cycle and regulating apoptosis [6, 7]. Therefore, the transcription of IE genes is a key point in the viral infection cycle. In recent years, the study on the promoter and transcriptional regulation of virus IE genes has become a research focus [8–10].

ICP46 was firstly found to encode a very early protein of approximately 46 kDa in frog virus 3 (FV3), and have been identified as IE genes in FV3, SGIV and Bohle iridovirus (BIV) [11–13]. By comparative genomic analysis, ICP46 is defined to be one of the Iridoviridae core genes and might play an essential role in the life cycle of iridoviruses [14]. It has been concluded that the transcription of FV3 ICP46 might be activated by certain virion-associated transcriptional transactivator (VATT) and depressed by at least one of the early proteins [6, 12]. Whereas the molecular identities of VATT and their precise mechanism of function are unknown. Our previous study demonstrated that SGIV ICP46 is an IE gene associated with cell growth control and contributes to virus replication, the transcription of SGIV ICP46 starts at 2 h p.i. and shows a high transcription level throughout the SGIV infection cycle [11]. It also revealed that SGIV ICP46 is a structural protein in virus nucleocapsid and aggregates mainly in cytoplasm of host cells during infection [11]. So far, the promoter sequence and transcriptional regulation of SGIV ICP46 are still unclear.

In the present study, we revealed the core region of SGIV ICP46 promoter, investigated the regulation model of SGIV ICP46 promoter associated with viral protein, identified the VATTs that interact with the SGIV ICP46 promoter, and determined the binding site of VATTs on SGIV ICP46 promoter. This study is important for the further study of the transcriptional regulation mechanism of SGIV ICP46, and contributes to better understanding of the iridovirus transcription, ultimately helps to develop novel control strategies for iridovirus diseases.

## Methods

### Virus and cell cultures

Grouper spleen (GS) cells were cultured at 25 °C in Leibovitz’s L-15 medium containing 10% fetal bovine serum (Gibco-BRL, Grand Island, NY, USA). Singapore grouper iridovirus (SGIV, strain A3/12/98 PPD) was propagated on GS cell monolayers as previously described [15]. Preparation of the SGIV suspension and purified SGIV virion were performed as described by Qin [16], and SGIV stored at − 80 °C until use. For SGIV infection, cells were infected with SGIV at a multiplicity of infection (MOI) of 2 in the following experiments.

According to Huang’s method, UV-inactivated virus was treated by exposing SGIV suspension under a 30-W UV light source (10 cm distant) for 30 min at 4 °C [17]. Heat-inactivated virus was obtained by 55 °C water bath for 30 min. Inactivated SGIV were stored at − 80 °C until use.

### 5′-untranslated region (5′-UTR) analysis

Transcription start site (TSS) of SGIV ICP46 mRNA was determined using 5′ RACE System for Rapid Amplification of cDNA Ends (Invitrogen, Carlsbad, CA, USA) according to the manufacturer’s instructions. First strand cDNA was synthesized from 3 μg total RNA isolated at 12 h p.i. using a specific reverse primer (GSP1, 5′-CAA AGG GTG TGG CAA G-3′). After RNAase treatment and cDNA purification, an oligo (dC) tail was added at the 5′ end. The resulting product was PCR amplified with a universal primer containing a poly-dG sequence and specific oligonucleotides (GSP2, 5′-TTT CCG CAG TGT ACG AGT CC-3′; GSP3, 5′-GAC GCG CAA GAC ATT GTG AG-3′) for the first and second PCR respectively. The amplified fragment was cloned into plasmid pMD18-T (TaKaRa, Dalian, China) and analyzed by automated sequencing (Sangon, Shanghai, China).

### Bioinformatics analysis

The TSS and 5′-UTR of SGIV ICP46 were performed bioinformatics analysis. According to the DNA sequences flanking the TSS (− 300 to + 200), the core promoter elements were analyzed for the TATA-box (TATA), CAAT-box ((C)CAAT) and downstream promoter element (DPE) (RGWYV (T)). Moreover, about 200 nucleotides (nt) flanking sequence of translation initiation site (TIS) from SGIV ICP46 and other 13 iridovirus ICP46s were chosen from the genome data of the NCBI blast server (Table 1), multiple nucleic acids sequence alignment was performed using Clustal X 1.83 and edited by the GeneDoc program. The potential TATA-box, CAAT-box and DPE were noted through bioinformatics analysis or previous reports [12, 13] on fourteen iridovirus ICP46s.

**Table 1:**
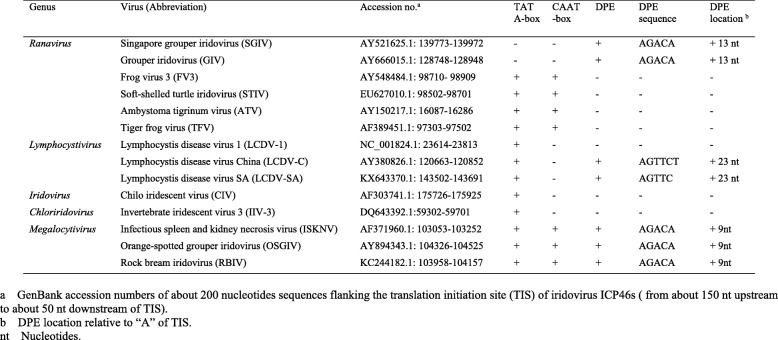
The core promoter elements analysis of iridovirus ICP46s

a GenBank accession numbers of about 200 nucleotides sequences flanking the translation initiation site (TIS) of iridovirus ICP46s (from about 150 nt upstream to about 50 nt downstream of TIS)

b DPE location relative to “A” of TIS

nt Nucleotides

### Plasmids construction of SGIV ICP46 promoter

Viral DNA was extracted from SGIV infected cells according to Xia’s method [18]. A luciferase reporter gene system was used to assay different lengths of SGIV ICP46 flanking region for promoter activity. According to the SGIV genome databases [19], with reference sequence ID (YP_164257.1), primers were designed to contain restriction sites for directional cloning as shown in Table 2. Recombinant plasmids were constructed by generating deletion mutants starting at positions − 3247, − 2180, − 1535, − 1159 or − 649 nt for SGIV ICP46 relative to the TSS. These promoter fragments were amplified by PCR from SGIV DNA using KOD-plus-Neo DNA polymerase (Toyobo, Osaka, Japan). The primers introduced KpnI restriction site at the 5′ end of the sequences. The primer at the 3′ end (Luc R1) annealed from position + 44 nt for SGIV ICP46 relative to the TSS and introduced a BglII restriction site. The amplified DNA fragment was digested with KpnI and BglII and cloned in-frame with a luciferase reporter gene in the vector pGL4.17 (Promega, Madison, WI, USA). In this way the promoter plasmids P-3247, P-2180, P-1535, P-1159 and P-649 were generated. Other 9 serially deleted promoter plasmids for SGIV ICP46 (− 559, − 502, − 442, − 360, − 279, − 196, − 136, − 82 and − 22) were amplified by PCR from the P-649 promoter plasmid. The primers directed against the 5′ end had KpnI restriction sites as described above. At the 3′ end, a primer (Luc R2) was used that annealed from position + 42 nt for SGIV ICP46 relative to the TSS. The resulting DNA fragments were cloned between the KpnI and BglII sites of pGL4.17, thereby generating P-559, P-502, P-442, P-360, P-279, P-196, P-136, P-82 and P-22 promoter containing plasmids. When the major determinant region for promoter activity was confirmed, a key-region-deleted reporter plasmid (P-Δ196) was constructed using primer Luc F-196 and Luc R3 (Table 2).

**Table 2:**
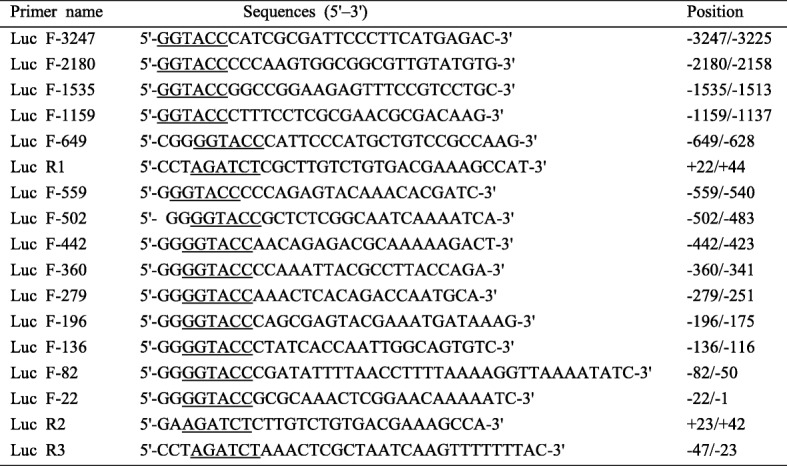
Oligonucleotides used for the preparation of the various promoter plasmids

Restriction sites were added to the gene specific SGIV ICP46 primers to assist cloning into the reporter vector pGL4.17; The underline shows the restriction enzyme KpnI and BglII

These different plasmids were sequenced for verification of the correct insert (Sangon, Shanghai, China). Plasmids were purified using a Plasmid Endo-free Mini Kit (Invitrogen, Carlsbad, CA, USA) and the DNA concentrations were determined by spectrophotometry (Nanodrop, Wilmington, DE,USA).

### Transfections and luciferase reporter assays

GS cells were seeded in 24 well plates at a density of 2.5 × 10^5^ cells per well in 500 μl of media and allowed to adhere overnight. All transient co-transfection experiments for luciferase reporter assays were carried out using 500 ng of each of the promoter reporter plasmids together with 50 ng of thymidine kinase promoter-Renilla luciferase reporter plasmid (pRL-TK) and Lipofectamine 2000 according to the manufacturer’s instructions (Invitrogen, Carlsbad, CA, USA). At 24 h after transfection, cells were infected with SGIV at MOI of 2 and further incubated at 25 °C. Cells were harvested at 6 h p.i. for promoter analysis. Firefly and Renilla luciferase activities were measured in cell extracts using the dual luciferase reporter assay system (Promega, Madison, WI, USA) following the manufacturer’s instructions.

### Promoter activity assay

To clarify the regulation pattern of viral proteins on the activity of SGIV ICP46 promoter, GS cells were transfected with P-22, which containing the critical promoter regions, and then the transfected cells were either mock infected or infected with infectious SGIV, heat-inactivated SGIV and UV-inactivated SGIV, respectively. The GS cells transfected with pGL 4.17 were served as negative control. Finally, the luciferase reporter assays were carried out.

To analysis whether the newly synthesized viral proteins can influence the activity of SGIV ICP46 promoter, GS cells were transfected with P-22 and then the transfected cells were pretreated with 50 μg/ml cycloheximide (CHX) or 100 μg/ml cytosine arabinofuranoside (AraC) for 1 h before and throughout the SGIV infection. CHX or AraC-pretreated cells were infected with SGIV at MOI of approximately 2, then harvested at 6 and 48 h p.i., respectively. The P-22 transfected GS cells infected with SGIV were collected at the same time points and served as negative control. The luciferase reporter assays were carried out following the manufacturer’s instructions.

### Electrophoretic mobility shift assay

To confirm that the SGIV proteins may bind with the core region of SGIV ICP46 promoter, the electrophoretic mobility shift assay (EMSA) were carried out. According to the core promoter of SGIV ICP46, the 64 nt EMSA probe named as probe X was design and synthesis, the sequence of probe X is 5′-GCGCA AACTC GGAAC AAAAA TCGCC CTTGT GGAAG ATTTA AAAAT GGCTT TCGTC ACAGA CAAG − 3′. The whole protein extraction from GS cells (GS), SGIV-infected GS cells (SGIV+GS), CHX-pretreated and SGIV-infected GS cells (CHX + SGIV), AraC-pretreated and SGIV-infected GS cells (AraC+SGIV) and purified SGIV virion (SGIV) were obtained using the Cytoplasmic Protein/Nuclear Protein Extraction Kit (Viagene Biotech, Changzhou, China) according to the manufacturer’s instructions. Then the EMSA were performed using Non-Radioactive EMSA Kits (Viagene Biotech, Changzhou, China) following the manufacturer’s protocol. Briefly, the whole protein extraction (5 μg) from GS, SGIV+GS, CHX + SGIV, AraC+SGIV cells and purified SGIV virion were incubated with biotin-labeled probe X in reaction buffers for 20 min at room temperature. Incubated with biotin-labeled probe NFκB, the nuclear extracts with activated NFκB was used as positive control and nuclear extracts without activated NFκB was used as negative control. Protein-DNA complex was separated from free oligonucleotide on 5.5% nondenaturant polyacrylamide gels, and then transferred to positively charged nitrocellulose membranes (Milipore, Bedford, MA) by wet trans-blot. After the transfer, the crosslink DNA on the membrane was immobilized in a UV linker (Stratagene Stratalinker 1800). Then the binding-membrane was visualized with streptavidin-horseradish peroxidase followed by chemiluminescent detection.

### DNA pull-down assays

DNA pull-down experiments were performed using Pulldown Kits for Using Biotin-Probes (Viagene Biotech, Changzhou, China) according to the manufacturer’s instructions. Briefly, the whole protein extraction (30–90 μg) from GS, SGIV+GS, CHX + SGIV, AraC+SGIV cells and purified SGIV virion were incubated with the biotin-labeled probe X (6–10 nmol) and non-specific competitors for 60 min at room temperature. Then the DNA/Protein binding mixture were bound to streptavidin-agarose beads by incubating 40 min at room temperature with gently shake, and washed three times to remove unbound DNA/Protein. Beads-bound proteins were dissociated by boiled for 5 min in a dissociation solution and separated by electrophoresis on a SDS-10% polyacrylamide gel. The differential protein bands were chosen and identified by NanoLC-ESI-MS/MS Analysis (ProtTech, Suzhou, China).

### Analysis of transient expression of VATTs on SGIV ICP46 promoter activity

The full length genes (SGIV ORF012, ORF039, ORF057 and ORF072) encoding four VATTs of SGIV ICP46 promoter identified by DNA pull-down assays were cloned into eukaryotic vectors pcDNA3.1/His A (Invitrogen, Carlsbad, CA, USA) using corresponding primers (Table 3). These different constructs were confirmed by restriction enzyme digestion and DNA sequencing, then named as pcDNA-012, pcDNA-039, pcDNA-057 and pcDNA-072. At 24 h post transfection of GS cells with above recombinant vectors, RT-PCR was used to verify their expression in GS cells. To value the effect of these VATTs on the activity of SGIV ICP46 promoter, the plasmids pcDNA-012, pcDNA-039, pcDNA-057, pcDNA-072 alone or 4 combined plasmids (pcDNA-012 + pcDNA-039 + pcDNA-057 + pcDNA-072) were co-transfected with P-22 and pRL-TK into GS cells, respectively. Vector pcDNA3.1/His A was used as negative control. At 24 h after the transfection, cells were infected with SGIV and harvested at 6 h p.i. for promoter analysis. The co-transfected cells without SGIV infection were also harvested at the same time for luciferase reporter assays.

**Table 3:**
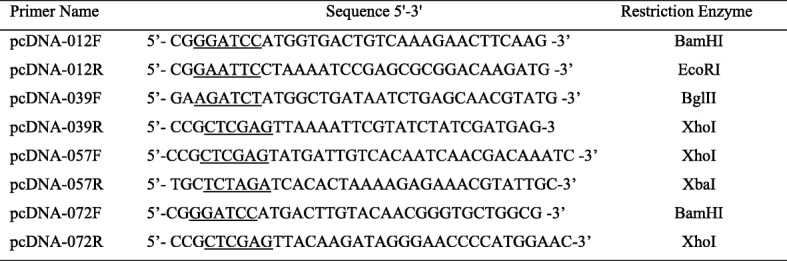
Primers used for the construction of recombinant eukaryotic expression plasmids

Restriction sites were added to the gene specific SGIV ICP46 primers to assist cloning into the eukaryotic expression plasmids pcDNA3.1/His A; The underline shows the restriction enzyme

### Determination of VATT binding site

To determine the VATT binding site on the SGIV ICP46 promoter, the 64 bp sequence of probe X were divided into five probes which were overlapped each other continuously (Table 4). After incubated with the protein extraction from purified SGIV virion, the EMSA were performed using Non-Radioactive EMSA Kits (Viagene Biotech, Changzhou, China) following the manufacturer’s protocol.

**Table 4:**
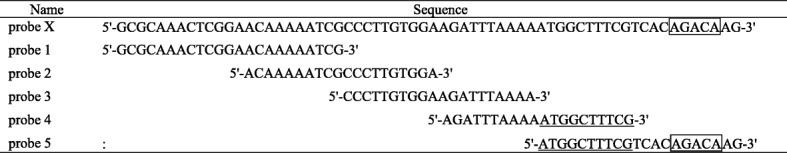
The probe used for defining the VATT binding site by EMSA

Framed sequence represent potential downstream core promoter element (DPE)

The determined binding site of virion-associated transcriptional transactivator (VATT) on SGIV ICP46 promoter are underlined

## Results

### SGIV ICP46 has short 5′-UTR and a potential DPE

**Fig. 1:**
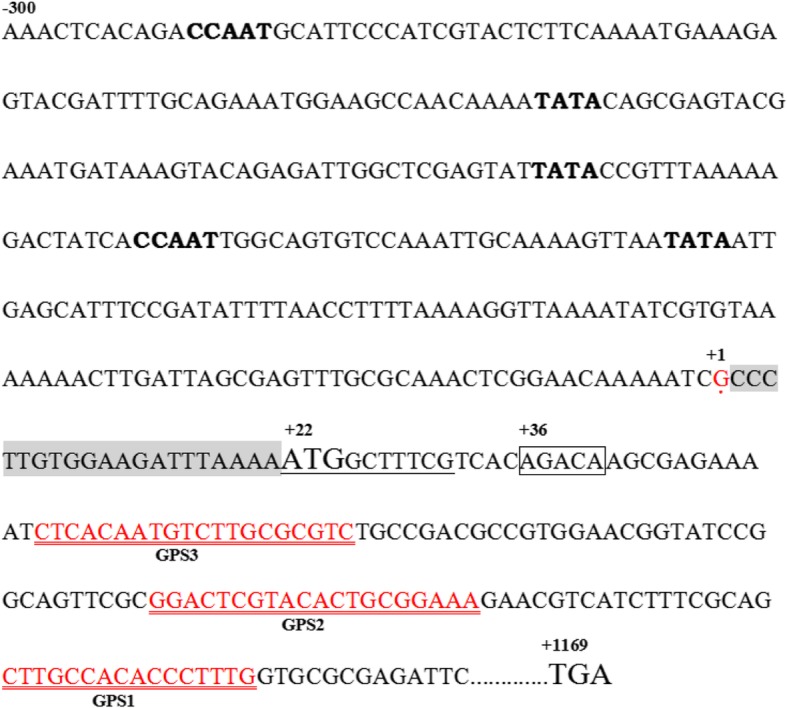
Determination of the 5′ end of the SGIV ICP46 gene transcripts by 5′ RACE analysis. The transcription start site (TSS) is position + 1. Sequence printed with shadow shows the 5′-untranslated region (5′-UTR). Primers used for 5′ RACE analysis are indicated as GSP1, GSP2, and GSP3 with double underline. Total ORF of SGIV ICP46 is 1149 nt. Bold sequences represent the TATA-box and CAAT-box like sequences. Framed sequence represent potential downstream promoter element (DPE). Underlined sequence show the binding region with virion-associated transcriptional transactivator (VATT)

**Fig. 2:**
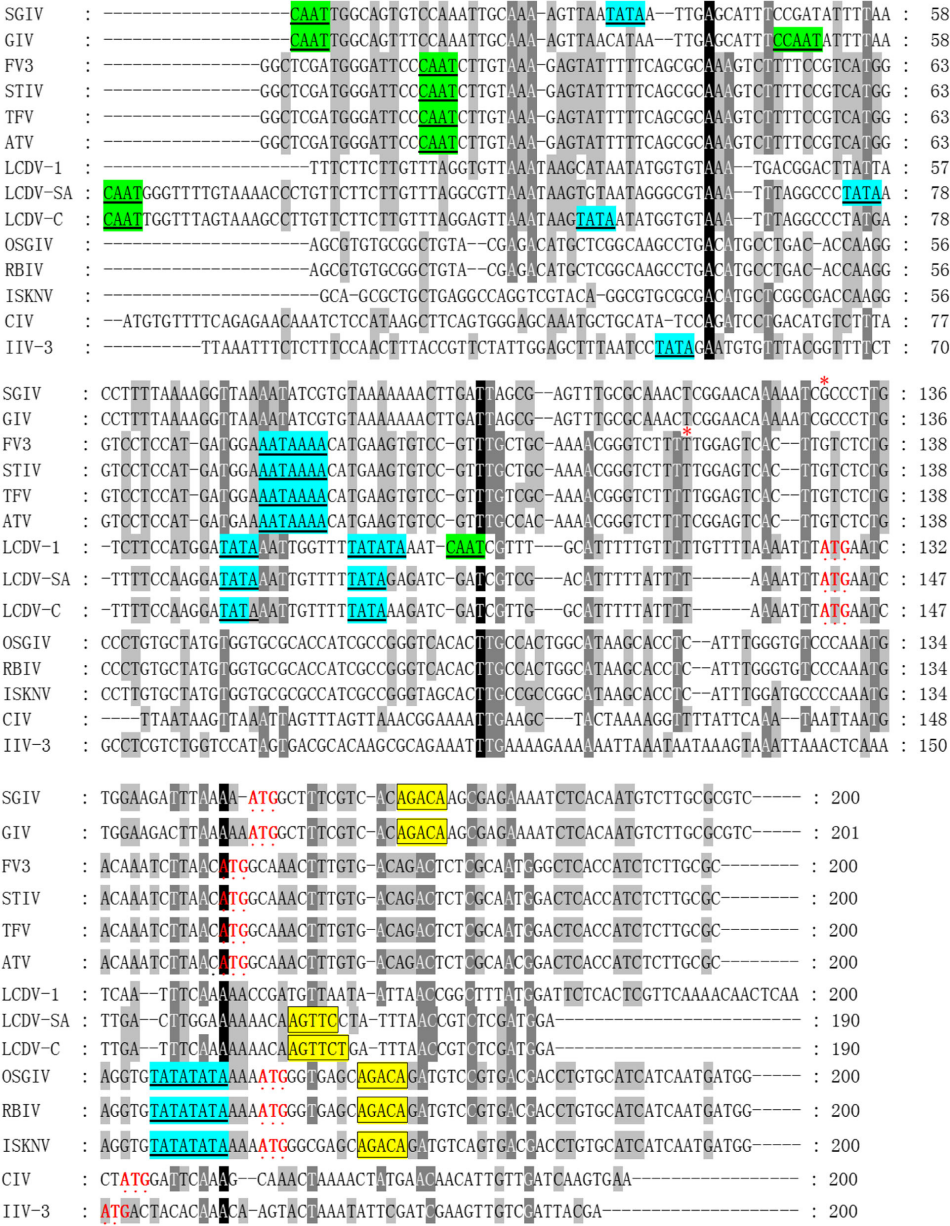
The core promoter elements analysis of SGIV ICP46 and other 13 iridovirus ICP46s. About 200 nucleotides (nt) flanking sequence of the translation initiation site (TIS) from SGIV ICP46 and other 13 iridovirus ICP46s were chosen, their GenBank accession numbers show in Table 1. Multiple nucleic acids sequence alignment of 14 iridovirus ICP46s was performed. The highly conserved residues in all sequences are indicated over a black background, sequences with high identities in one position are highlighted in dark grey or grey shadow. The TATA-box and CAAT-box like sequences are underlined. The the transcription start site (TSS) and the TIS are emphasized with symbol “*” and “·” respectively. Framed sequence represent potential downstream promoter element (DPE)

To determine the TSS of SGIV ICP46, 5′ RACE analysis was performed using total RNA extracted from GS cells at 12 h p.i. infected with SGIV. 5′ RACE was performed using three specific primers. PCR amplified fragments from 5′ ends were cloned and five individual SGIV ICP46 clones were sequenced. Data analysis showed that all sequences were identical and the TSS was at the G located 21 nt upstream of the TIS (Fig. 1). The 5′-UTR of SGIV ICP46 was found to be short and only 20 nt long. The core promoter elements analysis of SGIV ICP46 showed that no TATA-box was found within 94 nt upstream of TSS and no CAAT-box was found within 125 nt upstream of TSS. However a potential DPE, AGACA, was found at + 36 to + 39 nt downstream of the TSS (Fig. 1). According to the 200 nt 5′-flanking sequences of SGIV ICP46 and other 13 iridovirus ICP46s, three of the five genera of Iridoviridae were found to have presumptive DPE, they are *Ranavirus, Lymphocystivirus* and *Megalocytivirus* (Table 1). Senven presumptive DPE were found in SGIV, GIV, LCDV-C, LCDV-SA, ISKNV, OSGIV and RBIV (Table 1, Fig. 2). ICP46s of SGIV, GIV, ISKNV, OSGIV and RBIV did not contain a classical TATA-box at the putative location (about − 30 nt to TSS), while ICP46s of LCDV-C and LCDV-SA contained both TATA-box and DPE motif (Table 1, Fig. 2).

### SGIV ICP46 core promoter includes − 22 to + 42 nt to the TSS

**Fig. 3:**
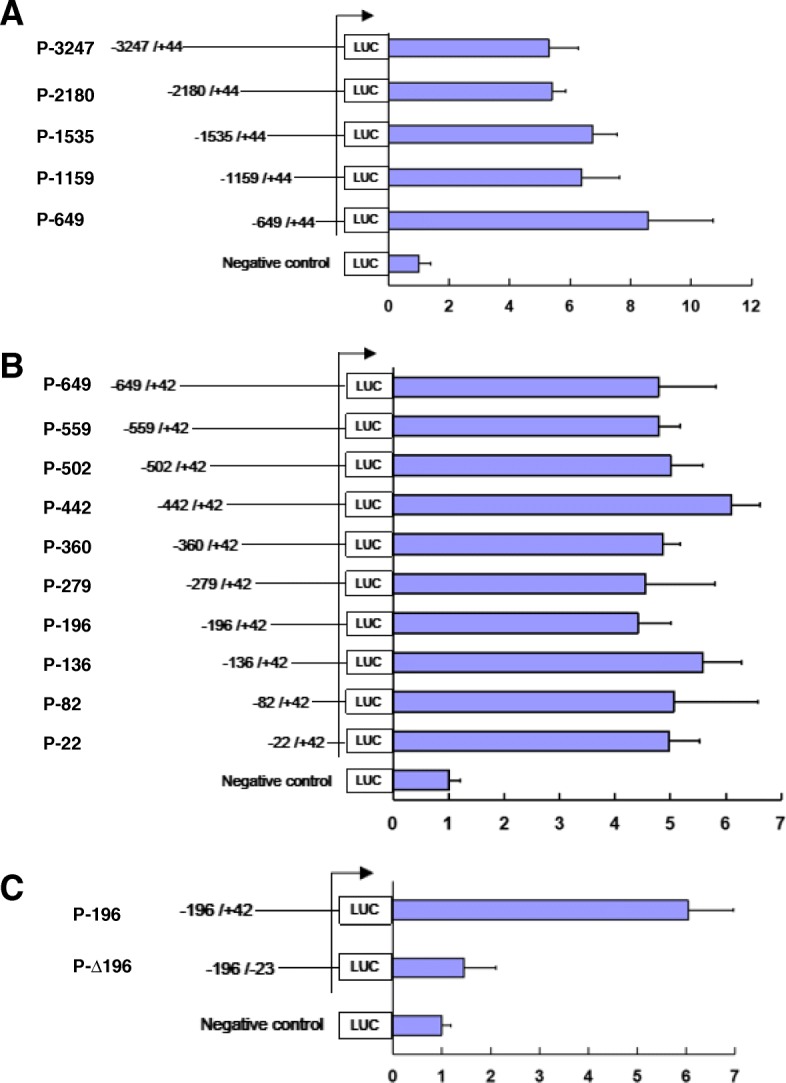
Deletion mutants of SGIV ICP46 promoters. **a** Luciferase reporter assays of consecutive deletion mutants from − 3247 to + 44. **b** Luciferase reporter assays of consecutive deletion mutants from − 649 to + 42. **c** Luciferase reporter assays of P-196 (− 196 to + 42) and P-∆196 (with − 22 to + 42 deleted from P-196). DNA fragments decreasing in length and located upstream of the open reading frames for SGIV ICP46 was fused to a luciferase reporter gene. GS cells were transfected with these promoter constructs followed by SGIV infection at MOI of 2 at 24 h after transfection. Cells were harvested 6 h p.i. for luciferase reporter assays. Firefly luciferase activities were normalized based on the activity of Renilla luciferase. The mean Fluc/Rluc intensity of negative control was considered as 1. The plasmid numbers on the left side specify the beginning and end positions (TSS as + 1) of deletion mutants. TSS are marked by arrows. Error bars show SD of three independent experiments

To identify the sequences important for the SGIV ICP46 promoter activity, a series of mutants with progressive 5′ deletions were cloned in front of firefly luciferase reporter gene. To prevent loss of promoter activity in case the promoter region would extend over the TIS, a small 5′ part of the open reading frame (ORF) of SGIV ICP46 (about 40 nt) was included as well. The resulting plasmids were transfected to GS cells together with pRL-TK as a control plasmid to correct for variations in transfection efficiency. Luciferase reporter assays showed that the luciferase activity was markedly increased after transfected the promoter reporter plasmids P-3247, P-2180, P-1535, P-1159 and P-649 comparing with the negative control plasmid pGL4.17, and the luciferase level of these five constructs had no significantly difference (Fig. 3a). The results indicated that the promoter region of SGIV ICP46 may locate between positions − 649 to + 44.

To further narrow the core promoter region, more promoter deletions (P-559, P-502, P-442, P-360, P-279, P-196, P-136, P-82 and P-22) were used for luciferase activity assay. The results revealed that even the shortest deletion of − 22 to + 42 still had the transcriptional effects with regard to luciferase activity (Fig. 3b), which demonstrated that a major determinant for SGIV ICP46 promoter activity is located approximately between positions − 22 and + 42.

After deletion of the key promoter region (− 22 to + 42 relative to the + 1 TSS) from P-196, the levels of luciferase activity of P-∆196 markedly reduced in SGIV-infected GS cells (Fig. 3c). This reduction in luciferase activity for P-∆196 confirmed that the 64 nt-region from − 22 to + 42 is the core region of SGIV ICP46 promoter.

### VATT are needed to activate the SGIV ICP46 promoter

**Fig. 4:**
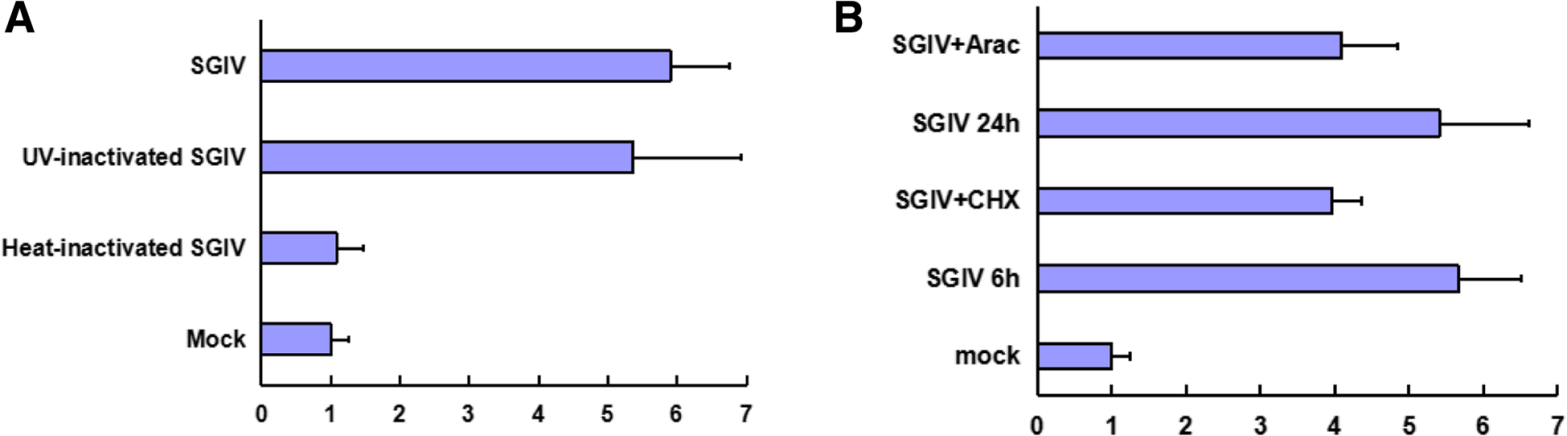
Regulation of the SGIV ICP46 promoter. **a** The transcription of SGIV ICP46 requires SGIV infection and is activate by heat-labile virion-associated transcriptional transactivator (VATT). GS cells were transfected with P-22, which containing the core region of SGIV ICP46 promoter, and then the transfected cells were either mock infected or infected with infectious SGIV, heat-inactivated SGIV and UV-inactivated SGIV, respectively. **b** CHX and Ara has no significant effect on the activity of SGIV ICP46 promoter. GS cells were transfected with P-22 and then the transfected cells were pretreated with cycloheximide (CHX) or cytosine arabinofuranoside (AraC) for 1 h before and throughout the SGIV infection. CHX or AraC-pretreated cells were infected with SGIV, then harvested at 6 and 48 h p.i., respectively. The P-22 transfected GS cells infected with SGIV were collected at the same time points and served as control

The heat-inactivated SGIV has denatured virion-associated proteins and replicable DNA, while the UV-inactivated SGIV has active virion-associated proteins and damaged DNA. Comparing with the mock-infected cells, luciferase activities were markedly increased after infected with the infectious SGIV and UV-inactivated SGIV in the P-22 transfected GS cells, while the luciferase activity of the P-22 transfected cells infected with heat-inactivated SGIV was similar with the mock-infected cells (Fig. 4a). These results suggested that the transcription of SGIV ICP46 required SGIV infection and the SGIV ICP46 promoter was activated by certain heat-labile VATT.

To analysis whether the newly synthesized viral proteins can influence the activity of SGIV ICP46 promoter, P-22 transfected GS cells were treated with CHX and AraC, respectively. The luciferase reporter assay showed that CHX and Ara had no significant effect on the activity of SGIV ICP46 promoter (Fig. 4b). CHX can block translation, allow only IE viral mRNAs to be synthesized and prevent the expression of SGIV E genes and L genes in host cells, while AraC is the virus DNA synthesis inhibitor and can restrain the the expression of SGIV L genes. The results indicated that no SGIV early proteins or late proteins involved in the regulation of SGIV ICP46 promoter.

### VATTs that interact with SGIV ICP46 promoter are VP12, VP39, VP57 and MCP

**Fig. 5:**
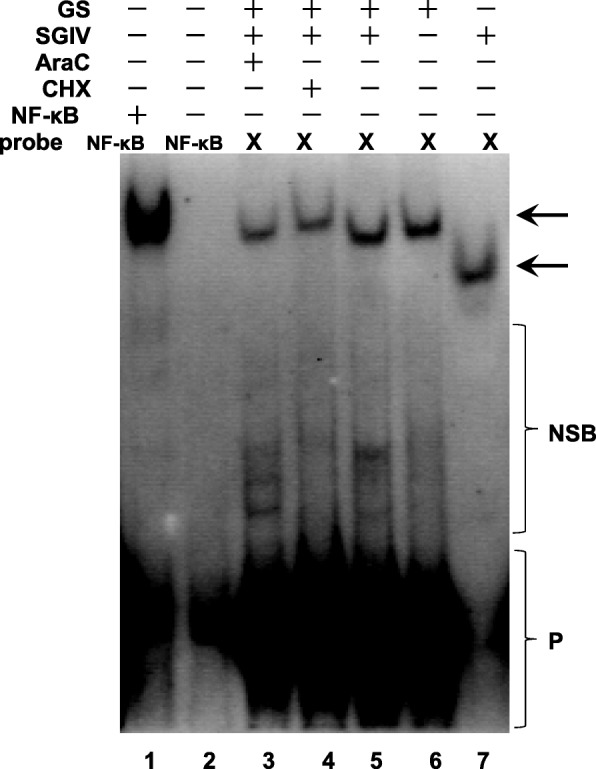
Electrophoretic mobility shift assay. The whole protein extractions from AraC-pretreated and SGIV-infected GS cells (AraC+SGIV, lane 3), CHX-pretreated and SGIV-infected GS cells (CHX + SGIV, lane 4), SGIV-infected GS cells (SGIV+GS, lane 5), GS cells (GS, lane 6) and purified SGIV virion (SGIV, lane 7) were obtained for EMSA analysis with biotin-labeled probe X that contains the sequence of SGIV ICP46 promoter. Incubated with biotin-labeled probe NFκB, the nuclear extracts with activated NFκB was used as positive control (lane 1) and nuclear extracts without activated NFκB (lane 2) was used as negative control. Arrow show the DNA binding proteins; NSB, non-specific binding; P, free biotin labeled probe

The EMSA probe, biotin-labled probe X, was design and synthesis according to the sequence of SGIV ICP46 promoter (− 22 to + 42). The protein extraction from GS, SGIV+GS, CHX + SGIV, AraC+SGIV cells and purified SGIV virion were used for EMSA analysis. The results show that all the protein extraction from GS, SGIV+GS, CHX + SGIV, AraC+SGIV cells and purified SGIV virion can combine with the probe X and form protein-DNA complexes. The band position of protein-DNA complexes from GS, SGIV+GS, CHX + SGIV and AraC+SGIV cells are basically the same, while the migration distance of protein-DNA complex from SGIV virion is further away from the sample holes (Fig. 5). The results suggested that the identified promoter region of SGIV ICP46 can interact with the transcription factors from both host cells and SGIV virion.

**Fig. 6:**
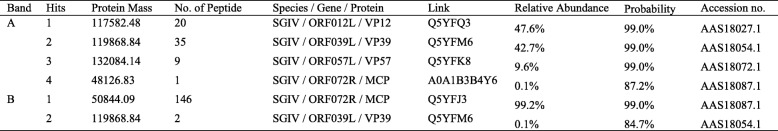
SDS-PAGE of DNA pull-down assays. DNA pull-down was performed with the biotin-labled probe X and the protein extraction from AraC+SGIV (lane 3), CHX + SGIV (lane 4), SGIV+GS (lane 5), GS cells (lane 6) GS, and purified SGIV virion (lane 7), respectively. Streptavidin-agarose bead (lane 1) was used as negative control and lane 2 is protein maker. Band A and B show the two DNA-binding proteins obtained in sample SGIV by DNA pull-down. Arrows show the protein bands choosed for NanoLC-ESI-MS/MS analysis

To Identify the VATT which may combine with SGIV ICP46 promoter and involve in the regulation of SGIV ICP46 promoter activity, DNA pull-down was conducted with the biotin-labled probe X and the protein extraction from GS, SGIV+GS, CHX + SGIV, AraC+SGIV cells and purified SGIV virion, respectively. The results of SDS-PAGE showed that the protein bands obtained by electrophoresis in GS, SGIV+GS, CHX + SGIV, AraC+SGIV cells were quite similar, and nine clear protein bands from SGIV+GS were selected for NanoLC-ESI-MS/MS analysis (Fig. 6 lane 5). For the protein extraction from purified SGIV virion, only two strong protein bands were observed (Fig. 6 lane 7). The mass spectrometry (MS) analysis showed that quite a few proteins from *Epinephelus coioides* were identified in the nine protein bands from SGIV+GS (data not shown), but no SGIV proteins were found might due to the low abundance. The MS data of the two DNA-binding protein bands (band A, B in Fig. 6) in SGIV virion sample are presented in Table 5, and four SGIV proteins that interacted with the SGIV ICP46 promoter were identified by MS. They are VP12, VP39, VP57 and major capsid protein (MCP) which are encoded by SGIV ORF012L, ORF039L, ORF057L and ORF072R, respectively.

**Table 5:**
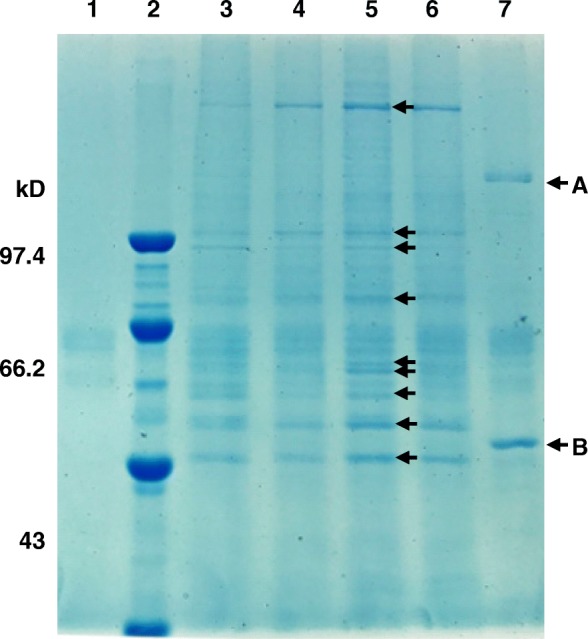
Identification of DNA-binding proteins that interact with the SGIV ICP46 promoter by MS

Protein Mass: Theoretical molecular weight of protein

No. of Peptide: Total number of identified peptide

Link: protein information link to https://www.uniprot.org/uniprot

Relative Abundance: Relative abundance of protein calculated by Label-free method

Accession no. GenBank accession numbers

### Effect of transient expression of VATTs on SGIV ICP46 promoter activity

**Fig. 7:**
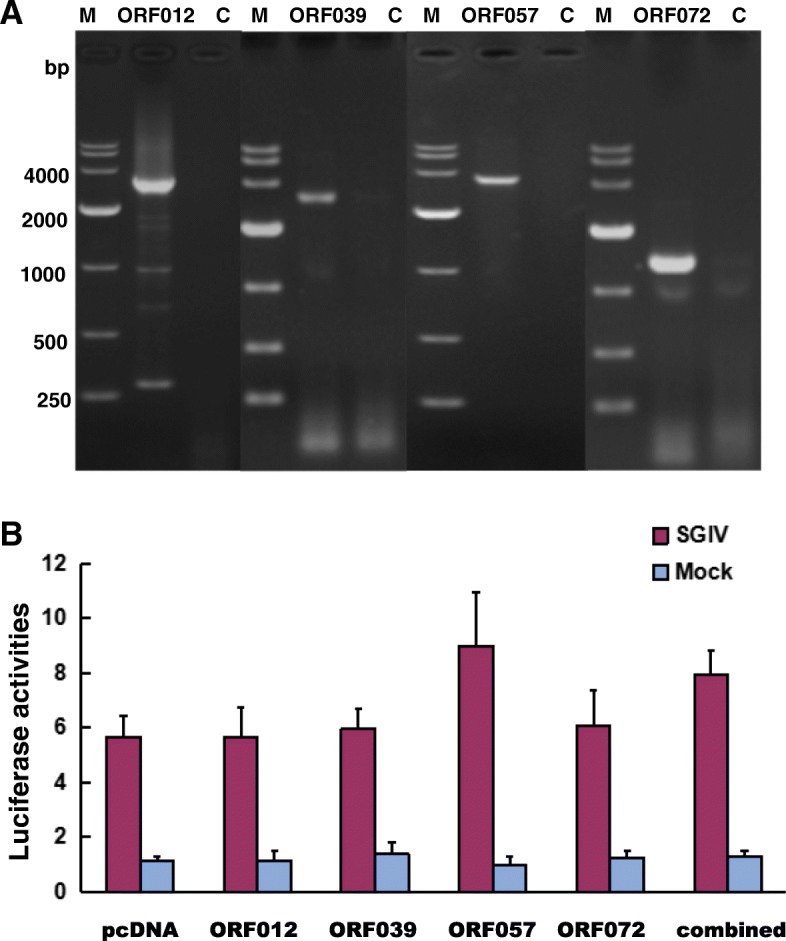
Effect on SGIV ICP46 promoter activity by transient expression of four VATTs. (**a**) Expression of four VATTs in GS cells verified by RT-PCR after transfected with pcDNA-012, pcDNA-039, pcDNA-057 and pcDNA-072 at 24 h post tansfection . lanes M, 10 kb DNA ladder; Lanes C, negtive control. (**b**) The luciferase reporter assays after co-transfection with pcDNA-012, pcDNA-039, pcDNA-057, pcDNA-072 alone or 4 plasmids combined. At 24 h post transfection, cells were infected with SGIV and harvested at 6 h p.i.. The co-transfected cells without SGIV infection were harvested at the same time for luciferase reporter assays. Firefly luciferase activities were normalized based on the activity of Renilla luciferase. The mean Fluc/Rluc intensity of negative control was considered as 1

To verify the effect of the four identified VATTs on the activity of SGIV ICP46 promoter, four recombinant eukaryotic expression vectors (pcDNA-012, pcDNA-039, pcDNA-057 and pcDNA-072) were constructed, and proved to be successfully expressed in GS cells by RT-PCR (Fig. 7a). In mock-infected samples, the luciferase activity exhibit similar low level after transfecting with pcDNA-012, pcDNA-039, pcDNA-057, pcDNA-072 alone or four plasmid combined comparing with the negative control plasmid pcDNA3.1/His A. The results showed that the expression of these four virus genes alone or in combination did not significantly affect the activity of the SGIV ICP46 promoter (Fig. 7b). Comparing with the mock-infected samples, the luciferase level increased markedly in all SGIV-infected samples. Together with SGIV infection, the luciferase activity in pcDNA-057 and combined plasmids transfected cells were slightly higher than that of pcDNA-012, pcDNA-039, pcDNA-072 and pcDNA3.1/His A transfected cells.

### The promoter binding site with VATTs is “ATGGCTTTCG”

**Fig. 8:**
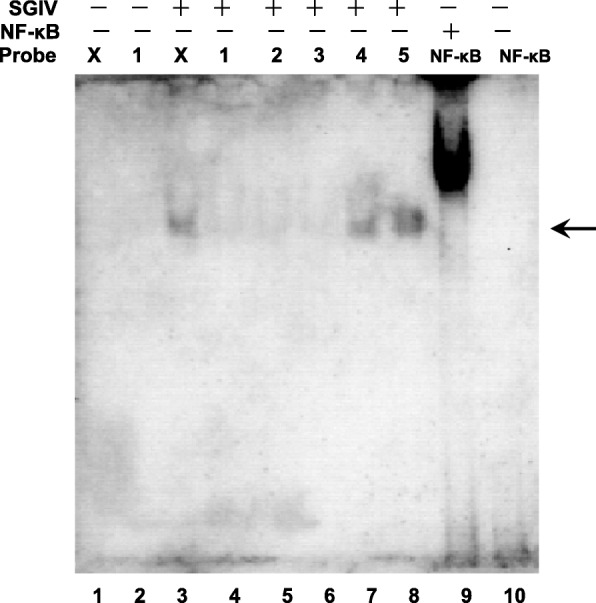
Determination of VATT binding site on SGIV ICP46 promoter by EMSA. Probe X that contains the 64 bp SGIV ICP46 promoter were divided into five probes which were overlapped each other continuously (probe 1–5 in Table 3). After incubated with the protein extraction from purified SGIV virion, the EMSA were performed. Arrow show the DNA binding proteins of SGIV. Incubated with biotin-labeled probe NFκB, the nuclear extracts with activated NFκB was used as positive control (lane 9) and nuclear extracts without activated NFκB (lane 10) was used as negative control

Since the VATTs are involved in the activation of SGIV ICP46 promoter, the binding site with VATTs of the SGIV ICP46 promoter was defined. Probe X and probe 1–5 (Table 4) were incubated with SGIV virion extracts respectively and EMSA were performed, the results show that the band of DNA binding protein of SGIV can be found in lane 3, lane 7 and lane 8 (Fig. 8), which mean that probe X, probe 4 and probe 5 can form protein-DNA complexes with SGIV VATTs. Probe X is 64 nt and has been proved can form protein-DNA complexes with SGIV VATTs (Fig. 5, lane7). Probe 4 and probe 5 are parts of Probe X and overlapped each other. The sequence of probe 4 is 5′-AGATTTAAAAATGGCTTTCG-3′ and probe 5 is 5′-ATGGCTTTCGTCAC**AGACA**AG-3′ (the overlapped part are underlined and the presumptive DPE displays in bold type). Taken together, it can be concluded that the overlapped squence of probe 4 and probe 5, ATGGCTTTCG, is the binding site with VATTs on SGIV ICP46 promoter.

## Discussion

The TSS of SGIV ICP46 was at the G located 21 nt upstream of the TIS. It has been proved that the RNA polymerases preferentially initiate transcription with a purine residue [20, 21]. Our study showed that SGIV ICP46 has a short 5′-untranslated regions (5′-UTR) of 20 nt. Previous studies revealed that transcripts in the Iridoviridae family may have short upstream 5′-UTR. For example the short 5′-UTR were found to be a common property for genes of Chilo iridescent virus (CIV), the CIV ORF012, CIV MCP and CIV DNApol genes have 35, 14 and 30 nt 5′-UTR, respectively [22, 23]. Furthermore, it was reported that the 5′-UTR were generally found to be short in FV3 [24]. The Bohle iridovirus (BIV) ICP 46, which promoter was identical to that of FV3 ICP46, has 30 nt 5′-UTR [13].

The location of typical TATA-box and CAAT-box is about − 30 and − 80 relative to the TSS respectively, while the core promoter elements analysis of SGIV ICP46 showed that no TATA-box and CAAT-box were found within 94 nt and 125 nt upstream of TSS, it seems that SGIV ICP46 promoter is a TATA-less and CAAT-less promoter. Meanwhile, a potential DPE was found at + 36 to + 39 nt downstream of the TSS. DPE is one of the core promoter element and plays an important role in the initiation of gene transcription by RNA polymerase II, the DPE functions cooperatively with the initiator (Inr) to bind to TFIID and directs accurate and efficient initiation of transcription in many TATA-less promoters [25, 26].

The DPE is usually located about 30 nt downstream of the TSS and most commonly found in TATA-less promoters [27]. It has been reported that the promoters of virus genes may contain DPE motif which is implicated in the control of promoter activity and gene expression. For example, the P38 of parvovirus and minute virus of mice (MVM) [28, 29], HSV-1 late genes [30], and the bro-c gene from *Bombyx mori* nucleopolyhedrovirus (BmNPV) [31] were all proved to contain DPE motif in their promoters. The bro-c gene of BmNPV has a pentanucleotide sequence CACGC located 30 nt downstream of the TSS and is essential for bro-c promoter activation. It reasonable to infer that the presumptive DPE of SGIV ICP46 may play an important role in the promoter activation and the initiation of gene transcription.

In addition, the core promoter elements analysis of 14 iridovirus ICP46s revealed that three of the five genera of Iridoviridae have potential DPE, they are *Ranavirus, Lymphocystivirus* and *Megalocytivirus*. Interestingly, *Ranavirus, Lymphocystivirus* and *Megalocytivirus* are exactly the three genera which can infect teleost fishes in family Iridoviridae. Seven fish pathogenic iridoviruses (SGIV, GIV, LCDV-C, LCDV-SA, ISKNV, OSGIV and RBIV) were found to have presumptive DPE. Although belong to the same genus *Ranavirus* as SGIV and GIV, no DPE were found in FV3, STIV, ATV and TFV, which are infectious to amphibians and reptiles. It is uncertain whether the presumptive DPE of ICP46 from fish pathogenic iridoviruses is associated with the transcription characteristic in teleost fishes. The DPE as core promoter elements for ICP46s of fish infectious iridovirus still need to be verified and studied in further research.

The promoter analysis found that the core regions of SGIV ICP46 promoter include sequence 22 nt upstream and 42 nt downstream of the TSS. Previous research about FV3 ICP46 showed that the promoter was from 291 nt upstream to 195 nt downstream of TTS, and found that the FV3 ICP46 was overexpressed in the presence of CHX and inferred that certain early proteins have negative control over FV3 ICP46 tanscription [12]. Different with FV3 ICP46, both CHX and Ara had no significant effects on the activity of SGIV ICP46 promoter, and indicated that the newly synthesized SGIV early proteins and late proteins in host cells did not markedly affect the activity of SGIV ICP46 promoter. Similar to FV3 ICP46, heat-labile VATTs were needed for activating the SGIV ICP46 promoter. No previous studies have identified the VATTs involved in ICP46 promoter activation. For the first time, four VATTs (VP12, VP39, VP57 and MCP) that can combine with the SGIV ICP46 promoter were identified by EMSA and DNA pull-down in this study. Coincidentally, VP12, VP39, VP57 and MCP have been identified to be virion-associated proteins in the SGIV viral proteome by 1-DE-MALDI and LC-MALDI workflow [32]. VP39, VP57 and MCP are Iridoviridae core proteins, and VP12 is a conserved iridovirus protein exist in genera *Ranavirus* and *Lymphocystivirus*. The function of VP12 is still unknown [19]. VP39 is a viral envelope protein and predicted to be a serine-threonine protein kinase, neutralization assays revealed that anti-SGIV VP39 serum could block SGIV infection [33]. Iridovirus MCP has been proved to be essential for virus replication by siRNA-based knockdown and anti-MCP serum-based neutralization test [34]. A homologue of SGIV VP57, CIV 295 L, is predicted to bind the viral DNA or DNA-binding proteins, has a function in the viral DNA replication or virus gene transcription [35]. Both SGIV VP57 and CIV 295 L have a predicted nuclear localization signal. The transcription of iridovirus IE genes are catalyzed by host polymerase II and VATTs may modify cellular polymerase II, interact with cellular transcription factors, or alter the DNA template and permit transcription of IE messages [3].

Co-transfection and luciferase reporter assays showed that the transient expression of four VATTs alone or combined did not significantly affect the activity of the SGIV ICP46 promoter without SGIV infection. The virion protein composition of five iridoviruses have been determined, including SGIV [32, 33, 36–38], these studies showed that iridovirus virions contain 40 to 64 virion-associated proteins and the protein-protein interactions between virion-associated proteins are required for viral assembly and other important functions throughout the course of infection [35]. It reasonable to infer that not only the presence of four VATTs but also the protein-protein interactions and structural connection existing among VATTs were needed for the transactivation of SGIV ICP46. The CIV interactome study have identified the interactions between virus structural proteins using the yeast two-hybrid system, and revealed that proteins 274 L and 295 L show indirect interactions with each other and protein 274 L is interacting with proteins encoded by seven different ORFs [35]. Interestingly, SGIV VP57 and SGIV MCP are just the homologues of CIV proteins 295 L and 274 L, respectively. Moreover, the virus promoters requires the synergistic activation of multiple transcription factors, including host and viral transcription factor, as well as various signaling pathway proteins interacting with these transcription factors [39–41]. Besides the four VATTs, many transcription factors from host cells also can combine with the SGIV ICP46 promoter and involved in the initation of gene transcription. Whether the network of protein-protein interactions existing among four VATTs of SGIV ICP46 promoter and the detailed mechanism about how VATTs activate the SGIV ICP46 promoter still need further studies.

The VATT binding site was defined by EMSA and reavealed that the 10-base DNA sequence “ATGGCTTTCG” between the TSS and the presumptive DPE is the binding site with VATTs. Transcription is a complicated process which involves the interactions of promoter elements with multiple transcription factors. The specific interactions rely not only on the specific sequence recognition but also on certain spatial arrangement of the factors in a complex [42]. The study about BmNPV bro-c promoter showed that the spacing between the TSS and DPE were also required for promoter activation [31]. On the SGIV ICP46 promoter, the 10-base DNA sequence “ATGGCTTTCG” is just between the TSS and presumptive DPE, and was proved to be the binding site of VATTs, it is reasonable to infer that the binding of VATTs with promoters helps to initiate the transcription of SGIV ICP46. In theory, interrupting the bind between VATTs and their binding site can effectively prevent the transcription of SGIV ICP46, then affect the temporal gene expression of SGIV and block the ordered cascade of viral life events, thus providing a new strategy for anti-virus infection.

## Conclusion

In conclusion, our present study found that the core region of SGIV ICP46 promoter locate from − 22 to + 42 nt relative to the TSS and probably is a TATA-less and DPE-containing promoter. This study also demonstrated that VATTs are required to initiate SGIV ICP46 transcription and the further molecular identities of VATTs are VP12, VP39, VP57 and MCP. Further examination revealed the binding site of VATTs interact with SGIV ICP46 promoter is ATGGCTTTCG between the TSS and presumptive DPE.

## Data Availability

Not applicable.

## Abbreviations

5′-UTR: 5′-Untranslated region
AraC: Cytosine arabinofuranoside
BIV: Bohle iridovirus
CHX: Cycloheximide
DPE: downstream promoter element
EMSA: Electrophoretic mobility shift assays
FV3: Frog virus
GS: Grouper spleen
IE: Immediate-early
MCP: Major capsid protein
MOI: Multiplicity of infection
MS: Mass spectrometry
SGIV: Singapore grouper iridovirus
TIS: Translation initiation site
TSS: Ttranscription start site
VATT: Virion-associated transcriptional transactivator
